# The Exposure of Phosphatidylserine Influences Procoagulant Activity in Retinal Vein Occlusion by Microparticles, Blood Cells, and Endothelium

**DOI:** 10.1155/2018/3658476

**Published:** 2018-07-03

**Authors:** Ying Su, Xueqing Deng, Ruishuang Ma, Zengxiang Dong, Feng Wang, Jialan Shi

**Affiliations:** ^1^Department of Ophthalmology, The First Affiliated Hospital, Harbin Medical University, Harbin, China; ^2^Health Ministry Key Laboratory of Cell Transplantation, Heilongjiang Institute of Hematology and Oncology, Department of Hematology, The First Affiliated Hospital, Harbin Medical University, Harbin, China; ^3^Department of Cardiology, The First Affiliated Hospital, Harbin Medical University, Harbin, China; ^4^Veterans Affairs Boston Healthcare System, Brigham and Women's Hospital and Harvard Medical School, Boston, MA, USA

## Abstract

The pathogenesis of hypercoagulability in retinal vein occlusion (RVO) is largely unknown. Whether the exposure of phosphatidylserine (PS) and microparticle (MPs) release will affect procoagulant activity (PCA) in RVO needs to be investigated. *Objectives*. To evaluate PS expression, circulating MPs, and the corresponding PCA in RVO patients. Twenty-five RVO patients were compared with 25 controls. PS-positive cells were detected by flow cytometry. Cell-specific MPs were measured by lactadherin for PS and relevant CD antibody. We explored PCA with coagulation time, purified coagulation complex assays, and fibrin production assays. In RVO, MPs from platelets, erythrocytes, leukocyte, and endothelial cells were increased and the exposure of PS was elevated significantly when compared with controls. In addition, we showed that circulating MPs in RVO patients were mostly derived from platelets, representing about 60–70% of all MPs, followed by erythrocytes and leukocytes. Moreover, PS exposure, ECs, and MPs in RVO lead to shortened clotting time with upregulation of FXa and thrombin formation obviously. Importantly, ECs treated with RVO serum which bounded FVa and FXa explicitly suggested the damage of retinal vein endothelial cells. Furthermore, lactadherin can inhibit the combination between PS and coagulation factors by approximately 70% and then exert an anticoagulant effect. In summary, circulating MPs and exposed PS from different cells may contribute to the increased PCA in patients with RVO. Lactadherin can be used for PS detection and an anticoagulant agent.

## 1. Introduction

Retinal vein occlusion (RVO) is a unilateral blinding disease including branch retinal vein occlusion (BRVO) and central retinal vein occlusion (CRVO). It was confirmed and demonstrated that people affected by RVO around the world were second to diabetic retinopathy [1]. The existence of retinal vein thrombosis may cause retinal capillary decompensation and even a sudden loss of vision without pain [2]. However, the pathogenesis of retinal vein thrombosis in RVO patients is not clear. Some genetically inherited factors associated with thrombosis including resistance to activated protein C and deficiencies of protein S and protein C have been studied in RVO patients [3–5]. Although there has been emerging evidence indicating that hypercoagulability and hypofibrinolysis are present in RVO patients [6], the molecular mechanism of RVO remains to be investigated. Some treatments against thrombosis such as isovolemic haemodilution, antiplatelet drugs, low molecular weight heparins, and fibrinolytic have been used with different results. However, none of them is totally effective, and a good therapeutic target achieved by a thorough knowledge of the coagulation is needed in order to treat the disease and improve visual acuity.

Phosphatidylserine (PS) is an anionic phospholipid, generally distributed in the cell membrane. When the cells are activated or upon apoptosis, PS will externalize to the outer membrane and combine with FV and FVIII to enhance the procoagulant activity (PCA) [7]. During this process, some membrane vesicles are named microparticle (MP) release. MPs range in size from 0.1 to 1 *μ*m in diameter and have different cellular origins like blood cells, endothelial cells, and other tissues [8]. Overproduction of MPs has been related to various physiological and pathophysiological conditions such as haemostasis and thrombosis, cell inflammation, angiogenesis, and apoptosis. MP formation was used to be considered a physiologic phenomenon; it was confirmed that increased levels of MP release and PS exposure are associated with a variety of diseases, such as diabetic retinopathy and nephrotic syndrome [9–14]. However, whether the PCA of the blood in RVO is mediated by MPs and PS has not been assessed.

Our aim was to explore the externalization of PS and MPs in RVO and further analyze their PCA. We investigated the MP release and PS exposure in RVO patients which may be important for the thromboembolic events of RVO. Lactadherin, a new sensitive probe and anticoagulant, can compete with PS for binding clotting factors V and VIII and was compared with annexin V in RVO patients [15]. Therefore, we used lactadherin to quantify PS^+^ cells and MPs in CRVO.

## 2. Materials and Methods

### 2.1. Study Subjects

This present study was permitted by each patient and agreed with the ethics committee of Harbin Medical University on the basis of the Helsinki Declaration. Enrollees were 25 patients newly diagnosed with RVO (10 CRVO and 15 BRVO) who were admitted between July 2015 and December 2016, compared with 25 healthy controls who were from a physical examination center, similar in age, sex, and associated pathologies. Except for the medical record, the ophthalmologist conducted a retinal angiography to collect ophthalmological data. RVO was diagnosed when the retinal area appeared superficial and there is a deep intraretinal flame-shaped haemorrhage. We should exclude the RVO patients who had accepted intravitreal ranibizumab, vitrectomy, or retinal laser treatment before. Some other factors affecting blood coagulation (exclude) are as follows: diabetes, hyperhomocysteinemia, antiphospholipid antibody syndrome, glaucoma, and so on (Table 1).

### 2.2. Preparation of MPs and MP-Depleted Plasma

Tubes filled with blood samples were centrifuged for the isolation of platelet poor plasma (PPP). PPP was carefully taken from the top of the tube, without disturbing the buffy coat, and centrifuged at 13000*g* for 2 min to gain platelet-free plasma (PFP). PFP was stored in liquid nitrogen immediately. Gradient centrifugation was used to get leukocytes. PFP was centrifuged for 45 min at 20,000 ×g to get MPs. Then the supernatant (MP-depleted plasma (MDP)) was washed with 25 *μ*l MPs.

### 2.3. Flow Cytometry Analysis of PS Exposure on RBCs, Platelets, WBCs, ECs, and Different Cell-Derived MPs

MPs (5 *μ*l) were stored in 35 *μ*l Tyrode's buffer and incubated for 10 min at room temperature in the dark Then it was analyzed by flow cytometry (FACS Canto II). To detect PS-positive platelets, erythrocytes, neutrophils, and endothelial cells, samples (50 *μ*l, 0.5–1 × 10^6^/ml) were incubated with Alexa 488-conjugated lactadherin^+^ CD41a (5 *μ*l), Alexa 488-conjugated lactadherin^+^ CD235a (5 *μ*l), Alexa 647-conjugated lactadherin^+^ CD45 (5 *μ*l), or Alexa 488-conjugated lactadherin^+^ CD31^+^CD41a^−^ (5 *μ*l). Flow cytometry (FACS Canto II) was used to test the exposure of PS on PlTs, RBCs, WBCs, and ECs binding with lactadherin. Platelet MPs (PMPs), RBC MPs (RMPs), neutrophil MPs (NMPs), and endothelial cell-derived MPs (EMPs) were defined by lactadherin^+^ CD41a^+^, lactadherin^+^ CD235a^+^, lactadherin^+^ CD66b^+^, and CD41a^+^/CD31^−^, respectively, and other cell origins by their specific monoclonal antibodies.

### 2.4. Endothelial Cell Culture and Reconstitution Experiments

Human umbilical vein endothelial cells (HUVECs) were cultured with endothelial cell medium (Science Cell, San Diego, CA, USA) at 37°C in a 5% CO_2_ humidified atmosphere. Endothelial cells (ECs) were dealt with media containing 20% of pool serum from 25 different RVO patients or donors at room temperature for 24 h. The PS exposure was defined by a flow cytometer. Each test was done in duplicate.

### 2.5. Coagulation Time and Inhibition Assays

The coagulation time of platelets, RBCs, WBCs, ECs, and MPs was detected in a STart4 coagulometer (Diagnostica Stago). One hundred *μ*l platelets (1 × 10^7^/ml), erythrocytes (1 × 10^8^/ml), leukocytes (1 × 10^6^/ml), endothelium (1 × 10^6^/ml), and MP suspension were incubated with 100 *μ*l MP-free human plasma for 180 s. One hundred *μ*l of 25 mM CaCl_2_ was added. For inhibition assay, 50 *μ*l lactadherin or anti-TF antibody was incubated with blood cells and MP suspension as before for 10 min. Clotting time was recorded.

### 2.6. Intrinsic, Extrinsic FXase and Prothrombinase Complex Formation and Inhibition Assays

Intrinsic factor Xase formation assay begins with the incubation of 10 *μ*l platelets (2 × 10^4^/ml), erythrocytes (2 × 10^5^/ml), leukocytes (2 × 10^4^/ml), endothelium (2 × 10^4^/ml), and MP suspensions with 1 nM factor IXa, 130 nM factor X (Enzyme Research Laboratories, South Bend, IN, USA), 5 nM factor VIII, 0.8 nM thrombin (Haematologic Technologies Inc., Burlington, VT, USA), and 5 mM Ca^2+^ in factor Xase buffer at room temperature. The reaction deals with the addition of EDTA. Subsequently, chromogenic substrates S-2765 (10 *μ*l, 0.8 mM) were added to each reaction system, and factor Xa product was determined immediately at 11 s interval for 15 min. For the extrinsic FXa formation assay, 2 × 10^4^ platelets were incubated with 130 nM FX, 1 nM FVIIa, and 5 mM Ca^2+^ for 5 min. The reaction was quenched by EDTA, and the amount of FXa formation was detected as described above. For prothrombinase formation assay, the samples were incubated with 1 nM factor Va, 1 *μ*M prothrombin (Haematologic Technologies Inc., Burlington, VT, USA), 0.05 nM factor Xa (Enzyme Research Laboratories, South Bend, IN, USA), and 5 mM Ca^2+^ at room temperature. Stop buffer EDTA and then 10 *μ*l Chromogenix S-2238 (DiaPharma Group, West Chester, OH, USA) were added into each microplate to evaluate the production of thrombin. Cells and MPs were dealt with varying concentrations of lactadherin (0–128 nM) or anti-TF antibody for 10 min in Tyrode's buffer. The mixture was dealt with the clotting factors. Then the production of FXase or prothrombinase was detected.

### 2.7. FVa/FXa Binding and Fibrin Formation on Cultured Endothelial Cell

FVa/Xa binding was observed with confocal microscopy; ECs were costained with factor Va-fluorescein-maleimide and factor Xa-EGRck-biotin (complexed to Alexa 647-steptavidin). Fibrin formation was quantified by turbidity in a SpectraMax 340PC plate reader [16]. ECs cultured on a 1% gelatin-coated coverslip chamber with RVO or normal serum were washed with Tyrode's buffer. Recalcified (10 mM, final) MDP (88% MDP, final) was added in the cultured endothelial cells.

### 2.8. Correlations between MPs and APTT and PT and FIB in RVO Patients

Correlations between MPs and APTT and PT and FIB were done using Pearson's correlation coefficients. The Spearman rank correlation was used to detect discrete variables.

### 2.9. Statistical Analysis

SPSS14.0 statistical software (SPSS, Inc., Chicago, IL, USA) was used for the analysis of each observation time point and each group. Data expressed as the mean ± standard deviation (SD) were made. Pearson's correlation coefficient was used. The comparison between different groups was analyzed by one-way ANOVA. The comparison between the two groups was analyzed using Student's *t*-test. *P* < 0.05 was considered statistically significant.

## 3. Results

### 3.1. Subject Characteristics

Clinical characteristics and blood laboratory results of retinal vessel occlusion patients and controls are presented in Table 1.

### 3.2. Microparticle Quantification by Flow Cytometry

**Figure 1:**
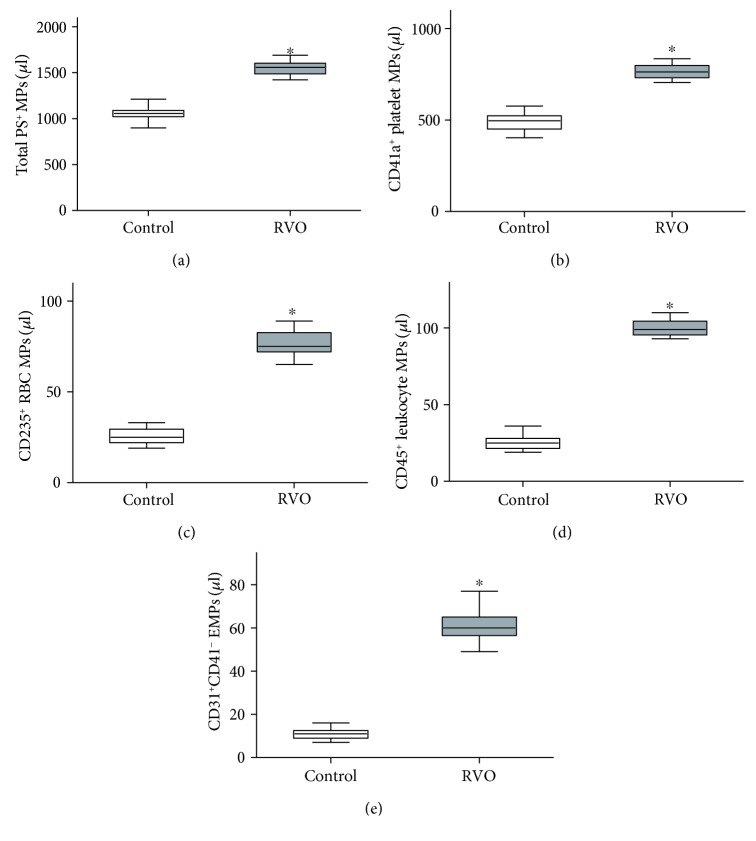
Flow cytometry analysis result of MPs in RVO. Box plots of (a) total PS^+^ MPs and their origin of (b) platelets, (c) erythrocytes, (d) leukocytes, and (e) endothelial cell were calculated (*n* = 25). MPs: microparticles; RVO: retinal vein occlusion. ^∗^*P* < 0.001 versus baseline.

The total number of MPs and their phenotypic characterization was measured (Figure 1). There is a significance of the total number of lactadherin^+^ MPs between the RVO group (1547 ± 83/*μ*l) and the controls (1058 ± 62/*μ*l, *P* < 0.001). Flow cytometry analysis result indicated that exposure of PS on platelets, red blood cells, leukocytes, and endothelial cells was significantly increased in retinal vein occlusion groups than controls (*P* < 0.001, Figure 1).

### 3.3. Phosphatidylserine Exposure on Cells

**Figure 2:**
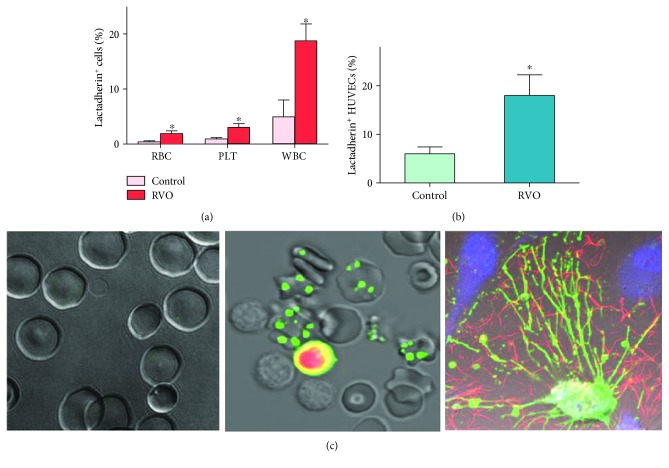
Flow cytometry and confocal microscopy of PS exposure on the plasma membrane. (a, b) Lactadherin-binding number of PLT, RBC, and WBC both in the control group (*n* = 25) and in the RVO group (*n* = 25) was measured. Data are displayed as the mean ± SD. ^∗^*P* < 0.001 versus controls. (c) Confocal microscopy images of normal erythrocytes (left), apoptotic erythrocytes and leukocytes in the RVO group stained with lactadherin (green) and PI (red) (middle) and ECs activated with RVO serum (lactadherin green; DAPI blue; fibrin red) (right) (*n* = 6).

The exposure of PS on the extracellular membrane of cells in healthy subjects and retinal vein occlusion patients was tested by flow cytometry (Figures 2(a) and 2(b)). Lactadherin-positive erythrocytes, platelets, and neutrophils were significantly elevated in the patients with RVO compared to those in controls (*P* < 0.05). The levels of MPs in RVO were detected because of PS exposure. There was a significant number of lactadherin^+^ MPs between the RVO group and the control group (*P* < 0.05). Lactadherin was also used to detect PS exposure of erythrocytes, leukocytes, and endothelial cells in each study group by confocal microscopy (Figure 2(c)).

### 3.4. Procoagulant Activities of Circulating Blood Cells and MPs

**Figure 3:**
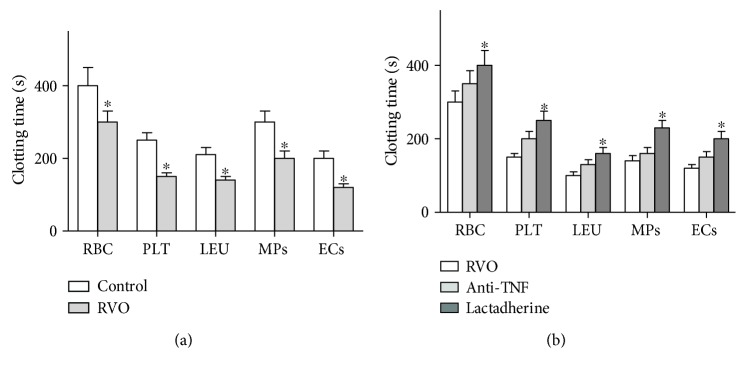
Coagulation time and inhibition assay. (a) Coagulation times of the control group and RVO patients were measured. Blood cells from RVO patients (*n* = 25) had more procoagulant activity than that from controls. ^∗^*P* < 0.05 versus control. Endothelial cells (ECs) in RVO serum showed more procoagulant activity (*n* = 10). (b) Coagulation times were inhibited about 70% by lactadherin (*n* = 10) (^∗^*P* < 0.05 versus anti-TF).

To detect the contribution of PS externalization to hypercoagulable state in RVO patients, PCAs of circulating blood cells and MPs were measured. The results showed that clotting times of erythrocytes, platelets, leukocytes, endothelial cells, and MPs were shorter in the RVO patients than in the control patients (*P* < 0.05, Figure 3(a)). To further explore the role of PS in the PCA of RBCs, PLTs, WBCs, ECs, and MPs in RVO patients, we performed coagulation inhibition assays. Lactadherin was used to block externalized PS, and the PCAs of RBCs, PLTs, WBCs, ECs, and MPs were markedly inhibited with lactadherin (*P* < 0.05, Figure 3(b)), while the anti-TF showed a little effect on the PCA of blood cells in RVO.

### 3.5. Formation and Inhibition Assays of Procoagulant Enzyme Complexes of Blood Cells, MPs, and ECs

**Figure 4:**
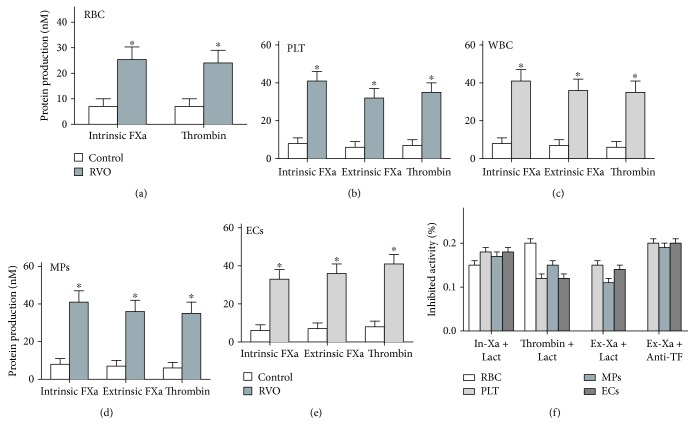
Formation and inhibition assays. FXa and thrombin production of 2 × 10^5^ red blood cells (RBCs) (a), 2 × 10^4^ platelets (PLTs) (b), 2 × 10^4^ leukocytes (WBCs) (c), 10 ml MPs from each group (25 controls, 25 RVO patients) (d), and 2 × 10^4^ endothelial cells (ECs) (e) is shown. Thrombin generation was examined in the existence of FXa and FVa. (f) The capacity of lactadherin (128 nM) to block procoagulant enzyme complexes on cells/MPs from 25 RVO patients or ECs incubated in RVO plasma was measured. ^∗^*P* < 0.001 versus controls.

To demonstrate the role of blood cells and MPs in hypercoagulability, purified factor tenase and prothrombinase complex generation assays were done. Platelets, erythrocytes, leukocytes, MPs, and ECs from RVO patients support the formation of individual enzyme complexes (Figures 4(a)–4(e)). The production of the procoagulant enzyme complexes elevated in the RVO group compared with the control group (*P* < 0.001). In inhibition assays, lactadherin (at 128 nM) blocked the production of the three procoagulant enzyme complexes by nearly 70% (Figure 4(f)); it is apparent that PS blockade almost entirely inhibits the complex formation, suggesting that PS independently increases the PCA in RVO.

### 3.6. PS Exposure on MPs and RVO Serum-Treated ECs Increase Prothrombinase Assembly and Improve Fibrin Formation

**Figure 5:**
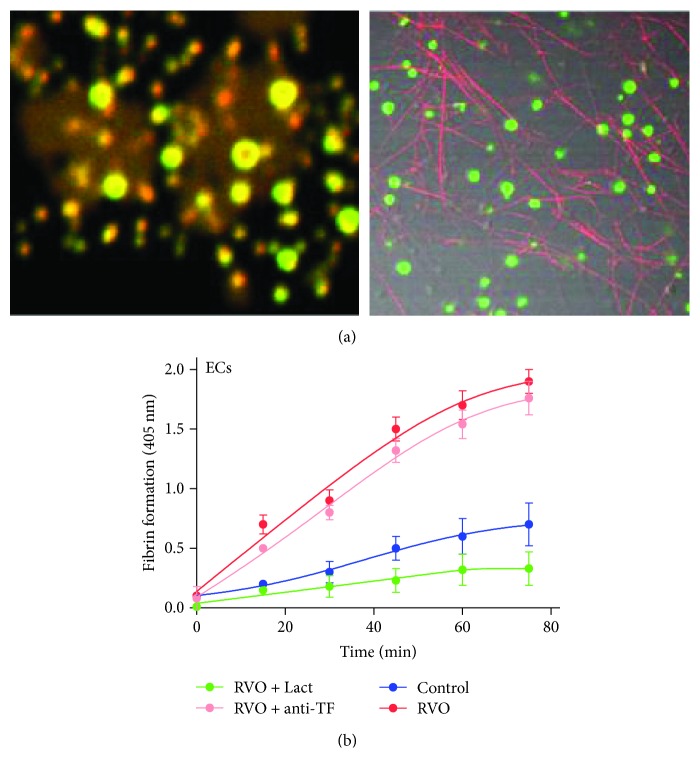
Endothelial cells (ECs) treated with RVO serum backing FVa/FXa binding and fibrin formation. (a) Confocal microscopy images of FVa/FXa binding (left, FVa green; FXa red) and fibrin formation (right, red) on the microparticles from ECs stimulated with RVO serum. (b) When compared with controls, RVO ECs resulted in significant fibrin production (*n* = 10). Lactadherin significantly inhibited fibrin formation.

We observed the FVa/FXa binding and fibrin formation in RVO serum-treated ECs. Cultured ECs were treated with plasma in the existence of calcium and fluorescence in labelled fibrinogen. Little change was observed when ECs were treated with pooled normal serum, while fibrin fibrils were distributed along the filopodia or cell margins of ECs rather than the whole cell surface. Little FVa and FXa were localized on ECs when treated with normal serum. We further observed the ability of ECs for fibrin formation. When compared with controls, ECs treated with RVO serum resulted in significant fibrin production. Lactadherin significantly inhibited fibrin formation, whereas anti-TF antibody did not significantly affect fibrin formation (Figure 5). Our results indicate ECs lead to PS-dependent fibrin production.

### 3.7. Circulating MPs Were Correlated with PT, APTT, and FIB in RVO Patients

**Figure 6:**
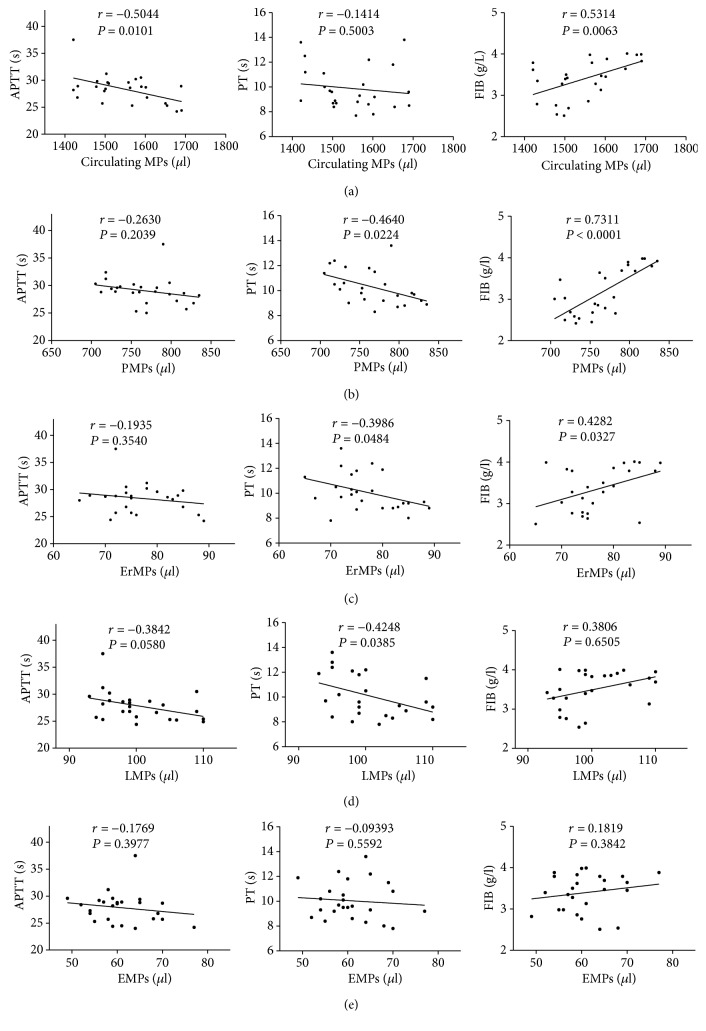
The correlation between MPs and APTT, PT, and FIB. Our result indicated that plasma levels of circulating MPs and other cell-derived MPs were significantly positively associated with the fibrinogen (FIB) in RVO. Plasma levels of circulating MPs and other cell-derived MPs were negatively associated with the APTT and PT in RVO (a, b, c, d, e). PMPs were strongly correlated with FIB (*P* < 0.0001).

We measured the parameters of coagulation, namely, activated partial thromboplastin time (APTT), prothrombin time (PT), and fibrinogen (FIB) in healthy subjects and patients with RVO. Our results revealed that circulating MPs (especially PMPs, *P* < 0.0001) were significantly related to FIB in patients with RVO (Figures 6(a)–6(e)). In addition, the level of FIB was remarkably upregulated in patients with RVO as compared with healthy subjects (Table 1).

## 4. Discussion

In our study, we first investigated the circulating MPs and their cellular origin in RVO patients. We showed that circulating MPs in RVO patients were mostly derived from platelets, followed by erythrocytes and leukocytes. Our results also revealed that PMPs were significantly correlated with FIB in patients with RVO. Moreover, we found the increased exposure of PS on the surface of PLTs, RBCs, WBCs, ECs, and MPs which provides binding sites for a lot of activated clotting factors including the FXa and prothrombinase complexes and thus promotes the coagulation cascade reaction leading to a dramatic increase in FXa, thrombin, and fibrin generation. Additionally, ECs treated with RVO serum-bound FVa and FXa explicitly suggested the damage of retinal vein endothelial cell. Lactadherin, which can bind with PS on PLTs, RBCs, WBCs, ECs, and MPs and block the production of the procoagulant enzyme complexes, plays an important role in the hypercoagulable state of RVO patients.

Previous studies focused on platelet activation and aggregation in RVO and have shown that *β*-thromboglobulin and platelet factor 4 are released distinctly in the plasma of RVO patients, which demonstrate that increasing platelets are activated and aggregated [16]. Moreover, Leoncini et al. found platelet hyperaggregability might be an important factor in the development of RVO due to their thrombogenic effects [17]. In our study, we found PMPs are the most abundant, representing about 60–70% of all circulating MPs in RVO. Increased levels of PMPs and PS^+^ platelets confirmed that platelets are activated. We then tested the potential correlation between the increased levels of circulating MPs and the hypercoagulable state. Our data revealed that the plasma levels of total circulating MPs and PMPs were positively associated with the level of FIB in RVO, indicating the procoagulant activity of circulating MPs in RVO. Interestingly, we further performed PS inhibition assays and proved that exposed PS on platelet-derived MPs supports the assembly of prothrombinase, leading to the generation of FXa and thrombin and formation of fibrin. Previous studies estimated that a platelet-derived MP generated in vitro has a 50–100-fold higher procoagulant activity than that on an activated platelet membrane [18], supporting our findings. Most studies found that antiplatelet therapy cannot prevent visual loss in the treatment of RVO patients [19–21]. However, the PCA of PMPs may play a more important role than platelets in RVO and more prospective randomized studies with an adequate sample size are needed to demonstrate the clinical benefit of antiplatelet therapy in RVO patients.

Substantial studies suggest that externalization of erythrocyte PS in SCD and abnormal CD239 (Lu/BCAM) phosphorylation on red blood cells from polycythemia vera (PV) patients may be crucial in these disorders [22, 23]. In this study, we demonstrated that levels of ErMPs and CD235a^+^ erythrocytes increased markedly in RVO patients than normal controls. Previous studies have reported that RBCs treated with RVO serum had increased PS on cell membrane [24], supporting our findings. Wautier et al. also found that when PS exposed on CRVO RBC combined with the PS receptor on an endothelial cell in static or flow conditions, it would induce hypercoagulable state and thrombophilia in retinal veins [25]. However, the effects of PS expression on other cells and the extent of PS exposure on different cells upon vein thrombosis in RVO are still unclear. Our studies supplied and extended the research above. It is well known that most studies focus on the association of leukocytes and the RVO patients with macular edema [26–28]; then our results confirmed firstly that PS-positive leukocyte-derived MPs (LMPs) and leukocytes are enhanced in RVO patients compared with controls. Leukocyte activation and inflammatory reaction will enhance the recruitment and aggregation, which then enhances the impairment of endothelial cells and blood-retinal barrier.

ECs also play an important role in maintaining optimal homoeostasis and blood fluidity within the intravascular space by balancing coagulation and fibrinolytic systems. Previous studies have found that the site-specific changes of vascular endothelial phenotype may be linked to the endothelium dysfunction in central or branch retinal vein occlusion and ECs are thought to be one of the key players in the pathogenesis of RVO [29, 30]. Notably, we demonstrated for the first time that ECs were treated with RVO serum binding FVa and FXa explicitly on filopods and fibrils of retracted ECs. The microvascular alterations and endothelial dysfunction may therefore result in retinal edema, hypoxia, ischemia, and angiogenesis, which subsequently develop into neovascular glaucoma and finally leading to visual loss. Some studies found an increased activation of clotting system and a decreased fibrinolytic potential in most patients with RVO. Moreover, increased levels of thrombin-antithrombin complexes (TAT) [31, 32], fibrinopeptide A (FpA) [33], and plasminogen activator inhibitor-1 (PAI-1) [34] were also found in acute retinal vein occlusion, supporting our findings. Collectively, the results obtained from our study confirmed that important venous endothelial changes and fibrin production suggest that endothelial-mediated PS exposure and MP release may be linked to the pathogenesis of RVO. In our study, lactadherin can remarkably inhibit the PCA of MPs and the related cells by reducing their procoagulant enzymes and fibrin production which can prolong coagulation time.

In summary, we illustrate the potential mechanisms of coagulation abnormalities in RVO that blood cells, endothelium, and circulating MPs participate in exuberant PCA via their PS exposure. More importantly, our results suggest the alterations in fibrin formation and deposition on endothelial cells in the pathogenesis of RVO. The differences in PS exposure and MP release cannot lead to cardiovascular risk factors because they were spread in the RVO and the control groups. The limitations of our study are the size of the population and the medical history of different lengths of time. PS inhibition assays demonstrate that the inhibition of PS might be a novel anticoagulant therapy strategy.

## Figures and Tables

**Table 1:** Characteristics of control subjects and RVO patients.

Baseline	Controls (*n* = 25)	RVO (*n* = 25)
Age (years)	58.72 ± 9.25	60.50 ± 10.25
Female sex, *n* (%)	15 (60)	18 (72)
Body mass index (median (kg/m^2^))	23 ± 10	26 ± 13
Dyslipidemia, *n* (%)	9 (36)	10 (40)
Hypertension, *n* (%)	10 (40)	10 (40)
Smoking, *n* (%)	14 (56)	15 (60)
Retinal vein neovascular	0	5
Macular edema	0	8
Visual acuity of involved eye, *n* (%)		
≤1/10	0	17
1/10 to ≤3/10	8	7
>3/10	17	1
Chronic open-angle glaucoma	0	0
Laboratory parameters		
RBC count (10^9^/l)	4.23 ± 0.83	4.54 ± 0.46
WBC count (10^12^/l)	5.60 ± 1.15	7.04 ± 2.89^∗^
Plt count (10^9^/l)	248.35 ± 50.32	260.62 ± 87.03
D-D (mg/l)	0.38 ± 0.14	0.46 ± 0.34
TT (s)	17.48 ± 1.13	18.12 ± 1.67
PT (s)	10.83 ± 0.70	11.09 ± 0.73
APTT (s)	27.14 ± 3.41	27.78 ± 5.50
Fib (g/l)	2.75 ± 0.55	3.40 ± 0.38^∗^

^∗^ *P* < 0.05 versus controls. RVO: retinal vein occlusion. D-D: D-dimers; TT thrombin time; PT: procoagulant time; APTT: activated partial thromboplastin time; Fib: fibrinogen.

## Data Availability

The data used to support the findings of this study are available from the corresponding author upon request.
